# Supplementary figures and images for: Volatile Composition and Sensory Properties as Quality Attributes of Fresh and Dried Hemp Flowers (Cannabis sativa L.)
Source: Foods. 2020 Aug 13;9(8):1118. doi: 10.3390/foods9081118 (PMC7466297; doi:10.3390/foods9081118)

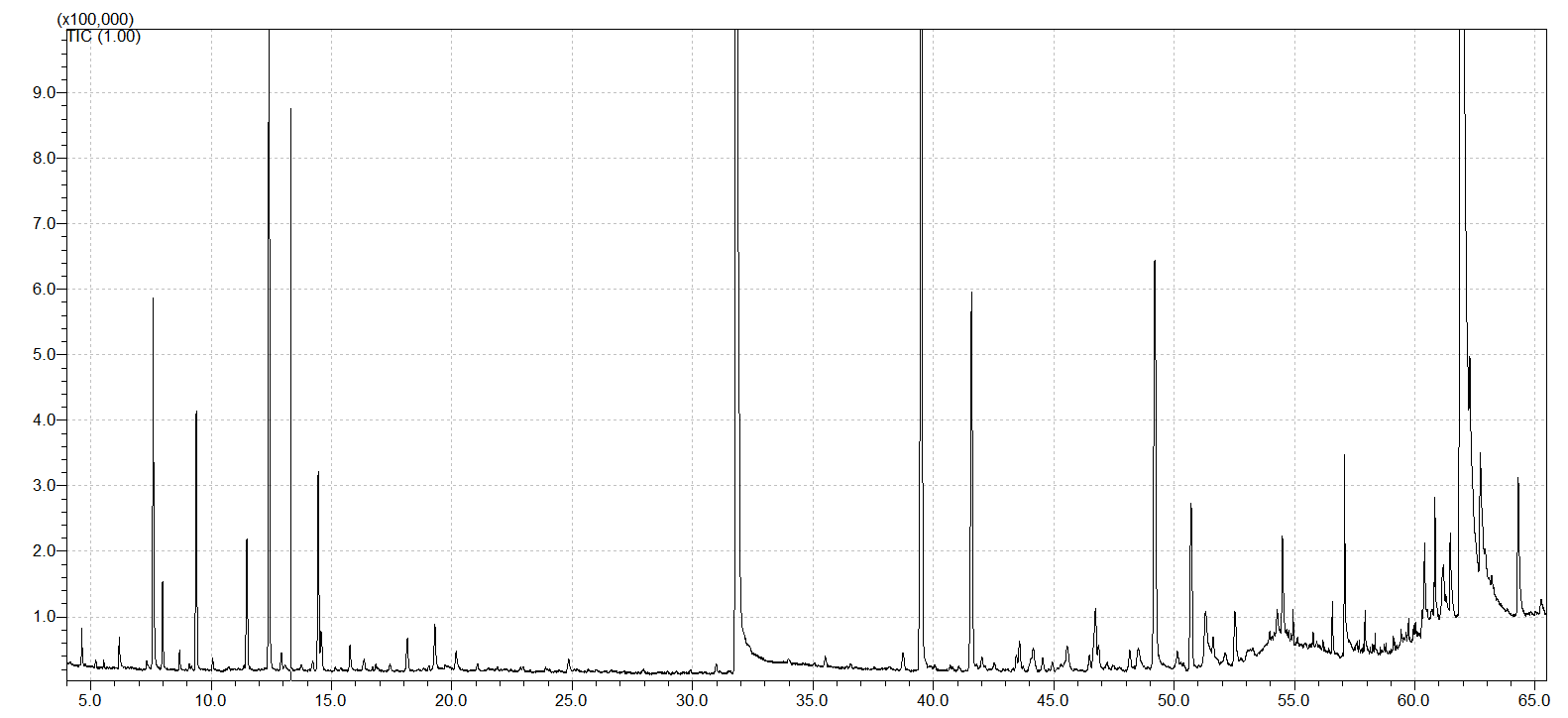

Supplement: Supplementary file 1 [file foods-09-01118-s001.zip › S1 Fresh hemp flower - GC-MS volatile profile analysis..png]

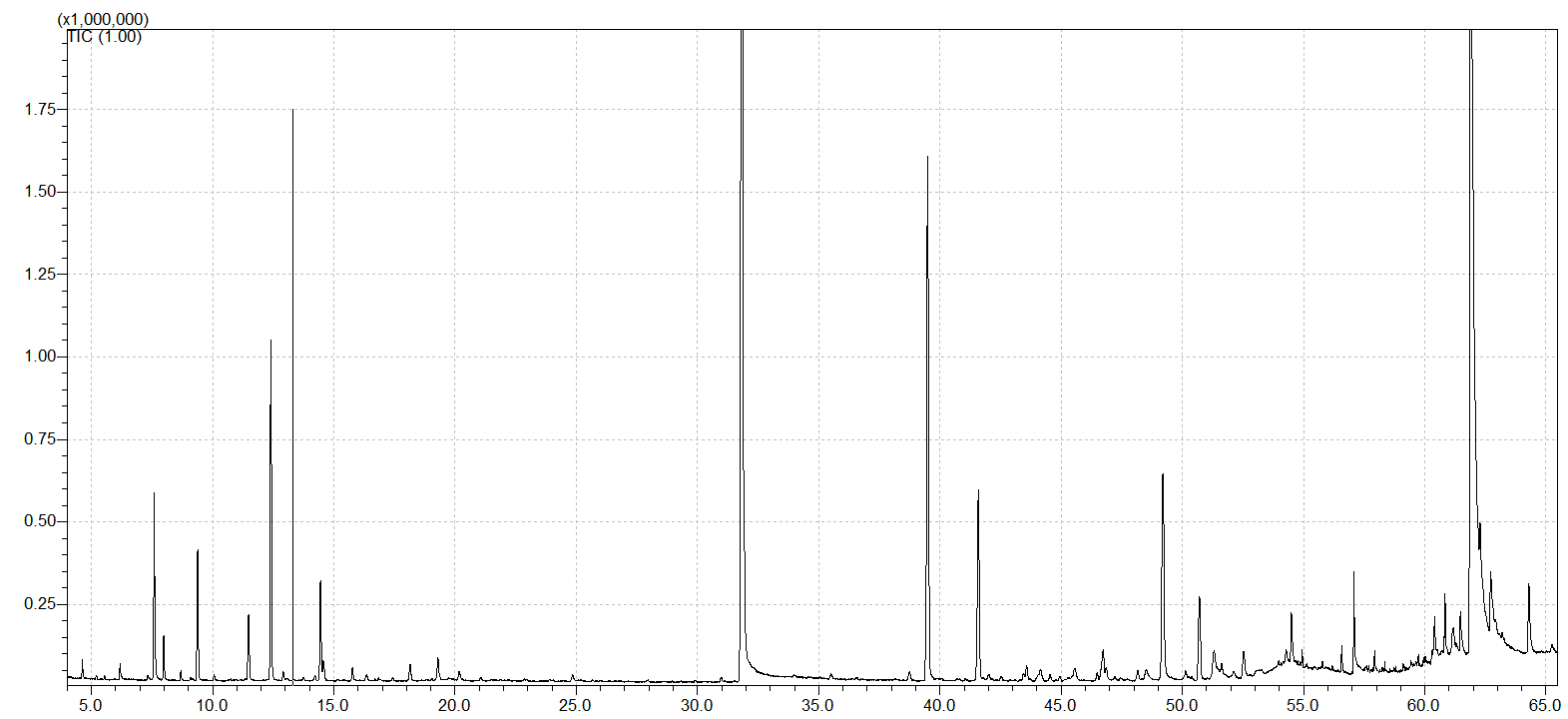

Supplement: Supplementary file 1 [file foods-09-01118-s001.zip › S2 Dried hemp flower at 240 W - GC-MS volatile profil analysis..png]
